# GryphSens: A Smartphone-Based Portable Diagnostic Reader for the Rapid Detection of Progesterone in Milk

**DOI:** 10.3390/s17051079

**Published:** 2017-05-10

**Authors:** Hyunwook Jang, Syed Rahin Ahmed, Suresh Neethirajan

**Affiliations:** BioNano Laboratory, School of Engineering, University of Guelph, Guelph, ON N1G 2W1, Canada; jangj@uoguelph.ca (H.J.); srahmed@uoguelph.ca (S.R.A.)

**Keywords:** progesterone detection, immunoassay, image analysis, point-of-care reader, android application

## Abstract

Enzyme-linked immunosorbent assay (ELISA) is a popular assay technique for the detection and quantification of various biological substances due its high sensitivity and specificity. More often, it requires large and expensive laboratory instruments, which makes it difficult to conduct when the tests must be performed quickly at the point-of-care (POC). To increase portability and ease of use, we propose a portable diagnostic system based on a Raspberry Pi imaging sensor for the rapid detection of progesterone in milk samples. We designed, assembled, and tested a standalone portable diagnostic reader and validated it for progesterone detection against a standard ELISA assay using a commercial plate reader. The portable POC device yielded consistent results, regardless of differences in the cameras and flashlights between various smartphone devices. An Android application was built to provide front-end access to users, control the diagnostic reader, and display and store the progesterone measurement on the smartphone. The diagnostic reader takes images of the samples, reads the pixel values, processes the results, and presents the results on the handheld device. The proposed POC reader can perform to superior levels of performance as a plate reader, while adding the desirable qualities of portability and ease of use.

## 1. Introduction

Point-of-Care Testing (POCT), as defined by the College of American Pathologists, is testing that takes place at or near the site where the patient is located and does not require dedicated laboratory facilities [1]. Scientific laboratories comprising controlled environments equipped with state-of-the-art equipment and tools can perform extensive tests on various subjects and samples and provide highly accurate diagnoses. Such facilities are, however, expensive to build and maintain and therefore remain in low numbers. People who live in remote areas or developing countries may have restricted access to emergency laboratory services. Even for routine services, accessibility and cost are often an issue. First, patients, samples, and livestock must often travel to the test site and back, which creates added transportation cost. Transportation may also need extra care for the patient or sample to prevent contamination or mutation risks, which also adds to transportation costs. Laboratory personnel are well-trained, and labor expenses for their services can be costly. Second, there is an additional pollution or contamination risk to the patient or sample anytime transportation is required, even with a high degree of protection and care. Third, when a central testing facility is used, waiting time is often added to processing time, which can create delays harmful to the patient or sample. The previous points are critical under urgent situations, such as an epidemic outbreak. Point-of-care testing solves many issues related to lab accessibility and expenses, simply by allowing tests to be performed near the site of origin at a reduced cost. Portable testing devices with simplified operating procedures will allow people with minimal training knowledge to perform diagnostic tests. There already exist powerful portable analysis devices that most people carry around daily: smartphones.

Smartphones, the pinnacle of modern personal electronics, have more processing power than most computers available a decade ago, while being portable and versatile. Apart from the original purpose of wireless communication, smartphones today come with numerous sensors, including accelerometers, gyroscopes, and magnetometers. Unlike traditional phones that only support cellular network communication, smartphones support numerous wireless communication protocols and specifications, such as IEEE 802.11 and Bluetooth for wireless local area network and communication over short distances, respectively. Every smartphone today runs on an advanced operating system that gives users and programmers flexibility with their own software design. Engineers and programmers have been developing smartphone applications that utilize hardware functionalities built into the smartphones to allow external devices to pair with them via a 3.5 mm audio port or a universal serial bus (USB) port, or wirelessly, using Bluetooth, Wi-Fi (Wireless Fidelity), or a cellular network. Apart from their technical features, smartphone devices are also an ideal platform for point-of-care diagnosis from an economic perspective. It is estimated there were 2.6 billion smartphone users in 2016 out of the world population of 7.4 billion [2]. This means that 35% of the world population owns smartphones. Therefore, smartphone applications for diagnostic purposes with easy-to-use user interfaces (UI) may turn them into a major healthcare monitoring device. While using a smartphone for health diagnoses may bring added convenience and increased accessibility in developed countries, it may have an even bigger impact in developing countries. Developing countries often have insufficient infrastructure, such as well-equipped laboratories and hospitals and poor transportation systems. Globalization and ecological pressures have increased the emergence of novel infections and global pandemics in farmed animals and humans. A key strategy for responding to such incidents involves ongoing monitoring and early detection of infection and outbreaks, but the population in developing countries may have less access to disease prevention and diagnosis. Concurrently, as developing countries grow economically, smartphone and mobile application markets are emerging, pushing manufacturers to produce more affordable devices. Therefore, smartphone-based POCT could aid in early diagnoses and disease prevention in developing countries.

The miniaturization and popularization of computers allows more people to integrate computers in various products and even in personal projects. Raspberry Pi, developed by the Raspberry Pi Foundation in the United Kingdom, is a Linux-based single board computer widely used for various purposes, including prototyping. The newest model, Raspberry Pi 3, has integrated Bluetooth and Wi-Fi functionality and requires no external dongle for connectivity. Raspberry Pi allows people to design low-cost prototypes, especially when combined with other devices, such as Arduino microcontroller kits and smartphones. As the internet of things (IOT) becomes a popular trend in device design and development, many companies are developing single board miniature Linux computers and providing a variety of products to designers and developers.

Progesterone (P4) is a sex hormone that affects the menstrual cycle, pregnancy, and embryogenesis in females of various species [3]. Measuring progesterone levels in cows’ milk helps predict the reproductive status in commercial dairy herds [4], detect oestrus or heat accurately [5,6], and diagnose early pregnancy [7]. Milk progesterone analysis is an efficient tool for troubleshooting causes of poor reproductive performance and can significantly aid livestock herd managers and veterinarians [8]. The advantages of milk progesterone analysis include the evaluation of response to hormonal treatments, differentiation of ovarian cysts, identification of open cows [9], and the monitoring of postpartum ovarian status [10]. There are no reliable on-farm tools available to test the concentrations of progesterone in milk. The cost of erroneous oestrus detection can be up to US $42 per cow during one cycle [11] and can amount to US $600 million annually [12]. To avoid economic losses and long calving intervals, precise oestrus detection through on-farm measurement of progesterone in milk is essential. It would greatly assist farmers to have a portable, inexpensive, and easy-to-use reader for progesterone detection and diagnosis. 

Gas chromatography [13], high-performance liquid chromatography (HPLC) [14], and mass spectrometry [15] are the analytical techniques used to determine the concentrations of progesterone in milk. These techniques require laborious sample preparation, high costs, and skilled personnel trained to use the benchtop equipment to generate the results. Read-out times, sensitivity, and specificity are some disadvantages of lateral flow technology [16,17,18] and ELISA kits [19,20], which have been used to detect progesterone in milk. Recently, Daems et al. [21] developed a Surface Plasmon Resonance (SPR) biosensor to detect progesterone in dairy milk as a competitive inhibition assay with a limit of detection of 0.5 ng/mL. However, SPR is expensive and is still a laboratory technique that requires integration with automated milking robots for efficient monitoring. There are no field-deployable sensors available for the on-site measurement of progesterone in milk.

The goal of this study is to enable and develop point-of-care testing for progesterone using a smartphone device and a Raspberry Pi portable reader, named GryphSens. A typical ELISA testing of progesterone in milk is performed, and pictures of the same milk sample used for ELISA testing are taken by the camera module in the GryphSens reader. GryphSens, which has an ultraviolet (UV) and infrared (IR) light emitting diode (LED), emits two types of excitation light with wavelengths of 395–400 nm and 850–950 nm, respectively. Milk samples with no progesterone were controls, and samples with known concentrations of progesterone were compared with the controls. OpenCV (Open Source Computer Vision) integrated in the smartphone application can generate histogram data of a picture, compare the histogram data, and output the similarities of different pictures with a double-formatted numeric value. The numeric value produced by OpenCV is then correlated with the ELISA testing result for analysis and validation. 

## 2. Materials and Methods

### 2.1. Progesterone Immune-Assay

The progesterone ELISA kit (PN 5081M, Abraxis Inc., Warminster, PA, USA) used for ELISA testing contains a 96-well microtiter plate, coated with a monoclonal anti-progesterone antibody, six 1-mL progesterone standard solutions (0, 0.23, 0.47, 0.94, 3.75, 20 ng/mL), two 1-mL control solutions (one low and one high concentration), one 11-mL enzyme conjugate solution, one 11-mL substrate solution, one 6-mL stop solution, and one 33-mL wash solution. All reagents were brought to room temperature prior to the experiment, and the 3 mL of wash solution was diluted at 1:15 v/v ratio with deionized filtered water (Milli-Q, Millipore Corporation, Billerica, MA, USA). Typical commercial 1% fat milk was diluted with Milli-Q water to various concentrations of 100%, 50%, 30%, 20%, and 10% for use as samples. The ELISA plate wells were coated with an antibody against progesterone and progesterone enzyme conjugate. Milk samples or standard solution were then mixed with a constant known concentration of an enzyme conjugate solution. Upon mixing the enzyme conjugation solution, incubation is required for 20 min followed by washing three times with the diluted washing solution. This was followed by addition of 100 µL of substrate solution to each well in the microplate and another 10 min of incubation. Finally, 50 µL of stop solution was added to each well and the Biotek Cytation 6 cell imaging multimode-reader (Biotek Instruments, Winooski, VT, USA) was employed at a wavelength of 450 nm with a reference filter at 620 nm. During the test period, progesterone and its enzyme conjugate competed to bind with antibodies on the surface of the wells. When the plate was washed and the substrate solution was added to each well, the solution turned blue with varying levels of saturation, depending on the progesterone concentration. Standard curves for progesterone P4 concentration provided by Abraxis were employed in the experiments as controls. 

### 2.2. Design of the Portable Diagnostic Reader and Smartphone Application

**Figure 1:**
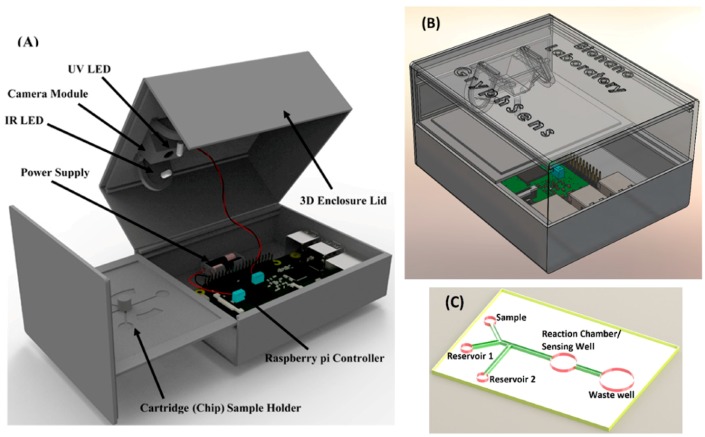
3D view of the developed GryphSens diagnostic sensor. (**A**) The detector comprises a sensor enclosure, 3D-printed lid, cartridge holder, and Raspberry Pi, UV and IR LED excitation sources, battery, and camera module; (**B**) 3D-rendered view of a fully assembled GryphSens diagnostic reader. The dimensions of the enclosed reader are 100 mm × 60 mm × 66 mm; (**C**). Schematic of the disposable analytical cartridge showing the microfluidic connections and reservoirs (not to scale).

The portable progesterone biosensor is based on an image processing test. In the digital domain, colors are represented, for instance, by a combination of 256 levels of red, green, and blue (RGB). Therefore, computer vision can distinguish 16,777,216 colors, and it can recognize different saturation levels in milk samples. This colorimetric test was performed with a portable diagnostic reader, based on the Raspberry Pi 3 model B (Raspberry Pi Foundation, Cambridge, UK), Pi NoIR Camera V2 (Raspberry Pi Foundation, Cambridge, UK), and an ultraviolet LED (400 nm peak wavelength, China Young Sun LED Technology Co., Shenzhen, China), and an infrared LED (940 nm peak wavelength, Vishay Semiconductors, Malvern, PA, USA) as excitation sources. An acrylic lens (AixiZ LLC, Houston, TX, USA), with a focal length of 8 mm, was used for magnification. The enclosure for the diagnostic reader was designed using the SolidWorks software (Dassault Systèmes, Vélizy-Villacoublay, France) and 3D-printed (Supplementary video 3). The enclosure ensured that every test was performed under a homogenous environment, the electronic components were held in place, and the sample and the camera were separated by the minimum object distance. Figure 1 shows the arrangement of the camera, the two LEDs, and the sample. Raspberry Pi 3 model B has 40 general-purpose input output (GPIO) pins for developers to turn on and off, and two pins were used to control the excitation LEDs using a 3.3 V TTL high input signal. Resistors of 680 ohms were used to set the intensity of the incident light excitation LEDs to ensure that the light from the LEDs fully covered the sample, while remaining safe from potential damage caused by overcurrent. A disposable analytical cartridge was also made of black polymers using a 3D printer. The cartridge dimensions are 40 mm × 30 mm with two reagent reservoirs (Figure 1C) and one reaction chamber linked by fluidic connections and was made of black Acrylonitrile butadiene styrene (ABS) polymer. The reaction chamber was designed to fit the dark box and the camera module. 

### 2.3. Software Development

In the presented instrument, Raspberry Pi on Raspbian Jessie, a Linux variation was specifically designed for Raspberry Pi. Python programming was used to control the Raspberry Pi camera. When an information string sent from a paired device arrives, the software parses the string, takes a picture with the selected excitation LED, and transfers the picture back to the paired instrument. A modification was made to the operating system to auto-start the Python software at boot-up. An Android smartphone application was developed for user interface and image processing. A Google Nexus 6P (Huawei Technologies Co. Ltd., Shenzhen, China) smartphone was selected for the development process, as it was the most recent Android instrument produced. The application was developed targeting Android software development kit (SDK) version 25, which runs on the newest Android Nougat OS and can support Android devices that use minimum SDK version 19, also known as Android KitKat. The user interface (UI) collected user inputs related to the test being performed, such as the name of the sample and the sample type. Also, it automatically fetches date, time, and location information for storage. Users can also select which LED to use for the excitation light by selecting a corresponding radio button. Screenshots representing the four activities of this application are given as Supplementary Materials (Figure S1).

### 2.4. System Architecture

Overall, the design that separates the sensor unit from the smartphone instrument ensures its performance is not affected by the model of the smartphone used, increasing compatibility while maintaining a constant performance level. Figure S2 shows the architecture of the entire system, from which the application and framework layers might be implemented on any smartphone instrument. The user interface is the front end of this architecture that interacts with users and the only component in the application layer. While the software has no functionality, a well-designed user interface [UI] is a crucial part of the system for user convenience.

In the framework layer, there is a computational unit and a communication channel. The computational unit performs every mathematical operation. In this specific design, the computational unit is mainly responsible for rendering the application, controlling the Bluetooth module on the Android device, handling events, gathering information, and analyzing the image taken by the Raspberry Pi module. The communication channel controls the data entering and exiting the computational unit. In this specific design, only Bluetooth is used for communication between the hardware layer and the application layer. However, modern smartphones support various communication protocols, which will be further utilized as development continues. The hardware layer represents the Raspberry Pi and the camera, as well as tasks performed within the portable instrument. Thus, it includes the Bluetooth module on the Raspberry Pi module and the input string parser from which the input data gets sorted out, and it controls the reader accordingly.

### 2.5. Sample Analysis Procedure

Prior to evaluating the performance of the portable diagnostic instrument, the progesterone content of the milk samples was measured using an ELISA assay to provide data that could be correlated to the outputs from the portable reader. Using the Cytation 5 cell imaging multi-mode reader (Biotek Instruments, Winooski, VT, USA), an absorbance spectrum was recorded using a wavelength sweep from 400 nm to 700 nm. After measurements on the plate reader were completed, images of the same samples were recorded under ultraviolet and infrared illumination and measured on the portable reader. To ensure the consistency of the image processing, an Android app was developed to crop the image from the Raspberry Pi reader and calculate the mean and standard deviation over all pixels present in the picture using a Java version of the OpenCV function from the core package.

### 2.6. Operating Parameters

The principle of the diagnosis lies in utilizing the relationship between the sample color and the progesterone concentration. The color camera senses light reflected off the sample surface. Tests were designed and conducted to evaluate the light intensity of samples as recorded by the camera, which varied from 85 µL to 125 µL under fixed UV and IR light intensity and had varying concentrations. Figure 1 shows the sample is well-designed to fit the portable reader. 

### 2.7. Statistical Analysis

Statistical analyses were conducted using Origin 8 (Origin Lab) software. Student’s t-test was used for comparing the test data of the obtained samples. The difference between the two sets of data was considered statistically significant with *p* < 0.05. Five independent experiments were conducted for each of the assays, and the mean value was used for plotting the graphs.

## 3. Results

### 3.1. Reader Assembly

There were two options to design the handheld reader. One was to process the image on the Raspberry Pi side, and the other option was to process the image on the Android smartphone. By comparing the CPU performance of each instrument, we concluded that the Nexus 6P Snapdragon 810 (Qualcomm, San Diego, CA, USA) has a higher processing power than the Raspberry Pi’s ARMv8 (ARM Holdings PLC, Cambridge, UK). The Nexus 6P has an Adreno 430 GPU (Qualcomm, San Diego, CA, USA) for graphics operation. It was therefore reasonable to select the Nexus for image processing and let the Raspberry Pi handle simpler tasks.

Figure 1 shows the 3D-printed enclosure and sample holder. It houses the Raspberry Pi, two excitation LEDs, three indicator LEDs, and a Pi camera. Two plates slide into the enclosure. The bottom plate is used to separate the Raspberry Pi compartment, the testing area, and the indicator LEDs. The top plate is used to hold the Pi camera and the excitation LEDs. The instrument is powered by a RAVPower 16,000 mAh external battery pack (RAVPower, San Jose, CA, USA) via the microUSB port, but it can be powered by any portable power bank that can provide 2.5 A of current. The camera, with the mounted lens, can fully capture the image of an object with a width and length of 0.7 mm. A sample holding well was designed so the wall creates no shadow on the sample surface and the wall edge is not seen by the camera. The overall form factor of the reader is 100 mm × 66 mm × 60 mm, and it weighs about 192 g; it can be carried easily to various test sites.

### 3.2. External Reference Progesterone ELISA Assay

**Figure 2:**
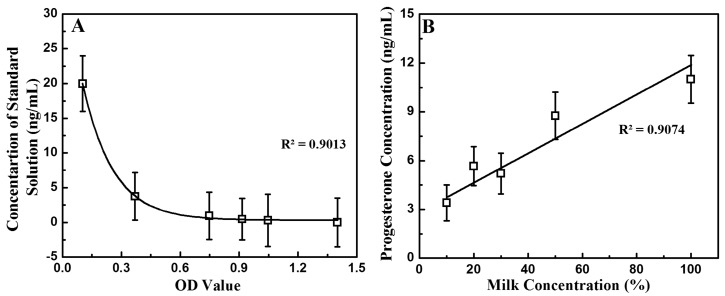
(**A**) Relation between the concentration of the standard solution and the measured optical density (OD) for the reference ELISA assay performed in the plate reader according to the Abraxis procedure; (**B**) Calibration curve between progesterone P4 concentration and the measured optical density (OD).

To assess the performance level of the developed instrument quantitatively, we performed a laboratory ELISA assay of the progesterone in the milk samples to set the standard of comparison. Thirteen samples were tested according to the Abraxis user manual procedure. The samples comprised six standard solutions, two control solutions, and five milk sample solutions diluted with water. Two duplicates of each sample were measured to ensure the accuracy of the reference values. The optical density (OD) values for each sample detected by the plate reader are given as Supplementary Materials (Figure S3). A simple relationship between the OD value and the progesterone concentration can be determined: the lower the progesterone concentration present in a solution, the higher its OD value. Since the progesterone concentrations in the standard solutions were known, a standard curve could be established, and the diluted milk samples were used as verification. Figure 2A shows the correlation between progesterone concentration and measured OD. In Figure 2B, a linear regression between the logarithm of the OD and the progesterone P4 concentration was performed to provide a calibration curve between the progesterone and milk concentrations of the sample. The following relationship was obtained with an R-squared value of 0.9013: *y* = −7.75 ln*f*(*x*) + 0.01. For reference, the progesterone concentration of each sample was 3.4 ng/mL, 5.6 ng/mL, 5.2 ng/mL, 8.7 ng/mL, and 11 ng/mL as the milk concentration in the sample rose from 10% to 100%. 

### 3.3. Portable Reader Testing

**Figure 3:**
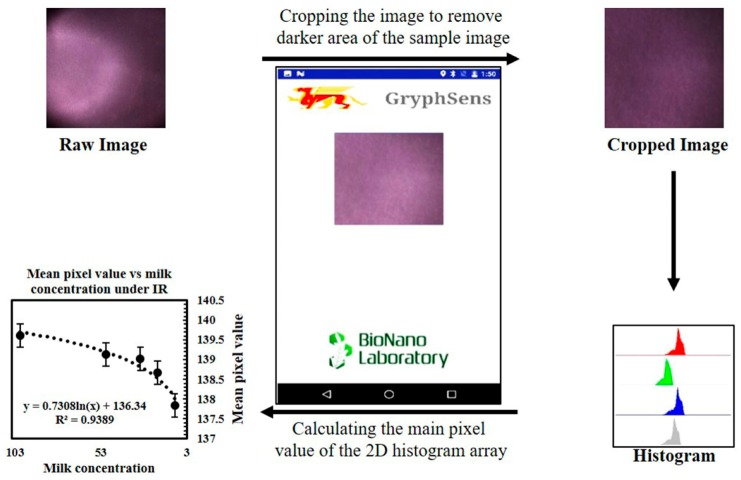
Flowchart illustrating the image processing procedure, including cropping, red, green, and blue (RGB) histogram extraction, and calculation of the mean pixel value.

Immediately after the conclusion of the ELISA assay, to avoid effects from sample deterioration, the same samples were measured on the portable instrument. The sample in each well from the plate reader was moved to a sample well designed for the portable reader, and three images using each excitation LED were acquired. Each image was then cropped, so only the portion where the sample color was constant would be processed. Figure 3 shows a flowchart of the image processing procedure. Using OpenCV’s predefined function, the mean value of the 2D pixel array was calculated and subsequently used to determine progesterone concentration in the corresponding sample. Similar to the ELISA assay, the results from the standard solutions were used to create a mean pixel value progesterone concentration standard curve. The progesterone levels in the milk samples were then plotted for comparison.

### 3.4. Optimization of Operating Parameters

**Figure 4:**
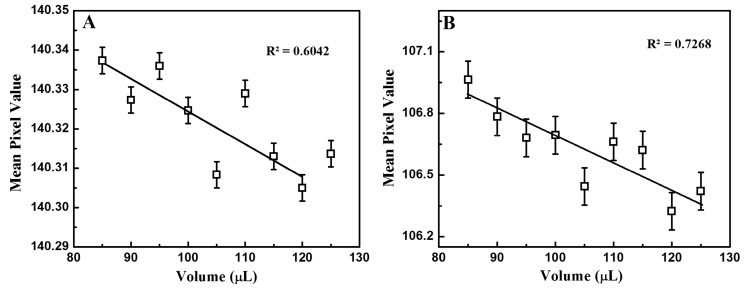
Dependence of the measured mean pixel value on sample volume under IR (**A**) and UV (**B**) illumination.

Sample volume is a parameter that affects sensor performance. Its influence was assessed to ensure the correct operation of the instrument. Figure 4 shows the correlation between the mean pixel value and a sample volume between 85 µL and 125 µL for both IR (A) and UV (B) illuminations. The standard solution at 0.23 ng/mL of progesterone was used for this measurement. A linear trend between the mean pixel value and the sample volume can be observed. The mean pixel value decreases as the volume of the sample increases, but occasionally deviates from the linear regression curve (R^2^ = 0.60 and 0.73, respectively). Since the mean pixel value for a 100-µL sample volume intercepts the linear regression fit under IR and UV illumination, all subsequent tests were performed with a volume of 100 µL, which seemed optimal for the signal-to-noise ratio in our reader.

### 3.5. Performance of Handheld Reader

**Figure 5:**
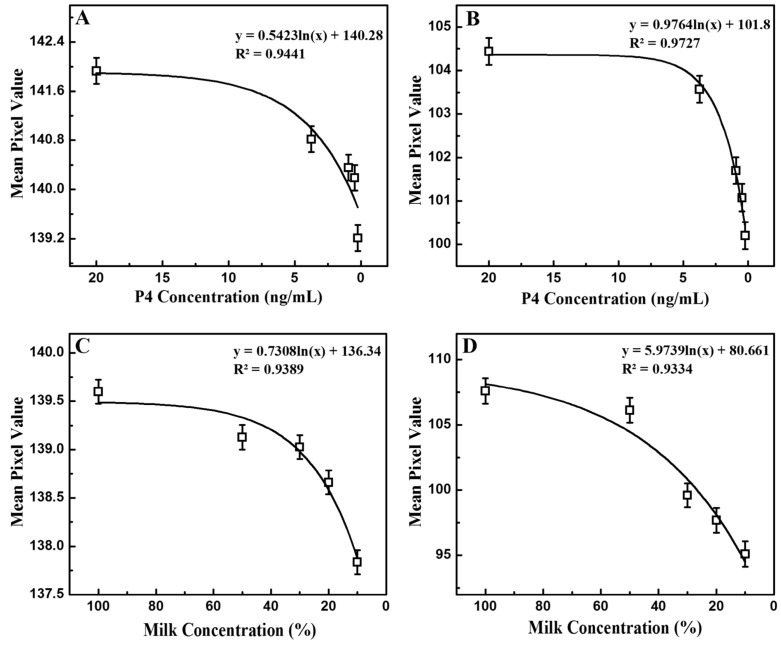
Evolution of the mean pixel value with progesterone P4 concentration in the standard solutions under IR (**A**) and UV (**B**) illumination and with milk concentration in the milk samples under IR (**C**) and UV (**D**) light.

**Figure 6:**
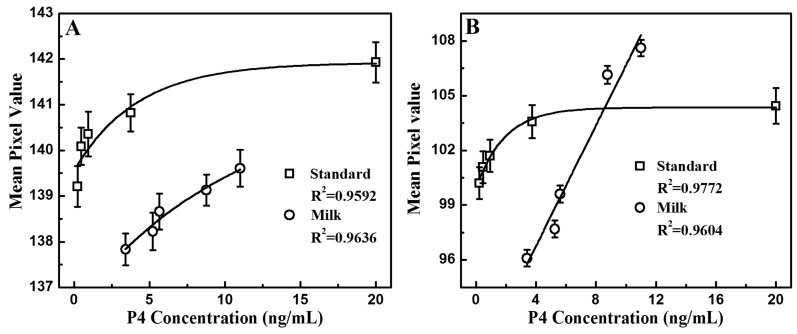
Differences between standard solutions and milk samples measured with IR (**A**) and UV (**B**) excitation.

To test the performance of the handheld reader, a similar procedure was used. Three images were acquired by the camera for each LED illumination, each image was cropped, and the mean pixel value was calculated for a sample volume of 100 µL as the sample concentration varied. The mean pixel values were averaged before being plotted. Figure 5 shows the evaluation of the standard solutions and the milk samples. Similar to the ELISA assay, where the OD was higher for samples with lower progesterone concentrations, Figure 5A and Figure 6B show that the mean pixel value of the image decreases as the progesterone concentration drops under both IR and UV light. Linear regression curves of *y* = 0.5423 ln*f*(*x*) + 140.28 with an R-squared value of 0.9441 and *y* = 0.9764 ln*f*(*x*) + 101.8 with an R-squared value of 0.9727 were obtained for IR and UV illuminations, respectively. There is a higher dynamic range in mean pixel value under UV light than under IR light. While the brightest and the darkest sample under IR only showed a difference of 2.722 mean pixels, the same samples under UV excitation displayed a mean pixel value difference of 4.236. Considering the progesterone detection using a plate reader is performed with light with a wavelength of 450 nm, it is expected that progesterone absorbs UV light better than IR light. For further analysis, the measurements were performed on milk samples (Figure 5C and Figure 6D) and a similar trend was observed. Two regression fits were obtained with R-squared values of 0.9389 and 0.9334, respectively. The samples again showed a bigger change in color under UV light than under IR illumination, which ensures the consistency of the results given by the reader. Figure 6 combines the results from the standard and milk solutions. Progesterone concentrations of milk samples obtained from the ELISA testing using the plate reader were used to generate Figure 6. There is a strong correlation with the results obtained from the plate reader, which indicates the validity of our measurements and the proposed POCT instrument. 

There are no cow side diagnostic kits or readers commercially available for real-time quantitative analysis of progesterone concentrations in milk. The levels of progesterone vary in individual samples of cow’s milk due to the correlation with the milk fat, and these levels also depend on the time of lactation and the oestrus cycle of dairy cows. The physiological progesterone levels in milk relates to the oestrus cycle, with highest levels during the luteal phase ranging from 0.2 to 30 µg/L, while for pregnancy, they range from 20 to 35.7 µg/L [22]. The limit of detection of our developed reader is 3 ng/mL, and is superior in performance compared to the commercially available semi-quantitative kits.

## 4. Conclusions

We have successfully designed, assembled, and validated a portable smartphone-based POC reader for the detection of progesterone in milk. The reader comprises UV and IR LEDs for illumination and a 3D-printed enclosure to provide all components with sufficient mechanical stability to ensure accuracy and a consistent performance level. In addition, a user-friendly Android smartphone application was developed to control all components of the POC instrument, acquire and process the sample images, extract the meaningful mean pixel value, and report its significance to the user. When compared with a standard ELISA assay for progesterone and a commercially available plate reader, the proposed POC instrument showed better performance levels, while clearly increasing portability and simplifying use, so it does not require trained laboratory personnel. Our POC reader provides the only available easy-to-use portable tool to farmers to monitor the progesterone content of the milk of their herd for various reproductive diagnostics, and it fills a void with a potentially high market impact. 

## Supplementary Materials

The following are available online at http://www.mdpi.com/1424-8220/17/5/1079/s1, Figure S1: Schematic of the software user interface (UI) for android smartphones to detect progesterone in milk samples, Figure S2: Overall system architecture and design of the portable progesterone detector in 194 smartphone-controlled biosensor devices. Figure S3: Representative data showing the optical densities (OD) and plate map of the 96-well plate used in the reader for the Abraxis reference ELISA assay.
