# Supplementary material for: GryphSens: A Smartphone-Based Portable Diagnostic Reader for the Rapid Detection of Progesterone in Milk
Source: Sensors (Basel). 2017 May 10;17(5):1079. doi: 10.3390/s17051079 (PMC5470469; doi:10.3390/s17051079)
Supplement: Supplementary file 1 [file sensors-17-01079-s001.pdf]

Supplementary Information

GryphSens: a smartphone-based portable diagnostic reader for the rapid detection of progesterone in milk

Hyunwook Jang, Syed Rahin Ahmed, and Suresh Neethirajan*

BioNano Laboratory, School of Engineering, University of Guelph,

Guelph, ON, N1G 2W1, Canada

*Corresponding authors. Tel: +1**-519-824-4120; Fax: +1-**519-836-0227

Email address: **sneethir@uoguelph.ca (S. Neethirajan)**

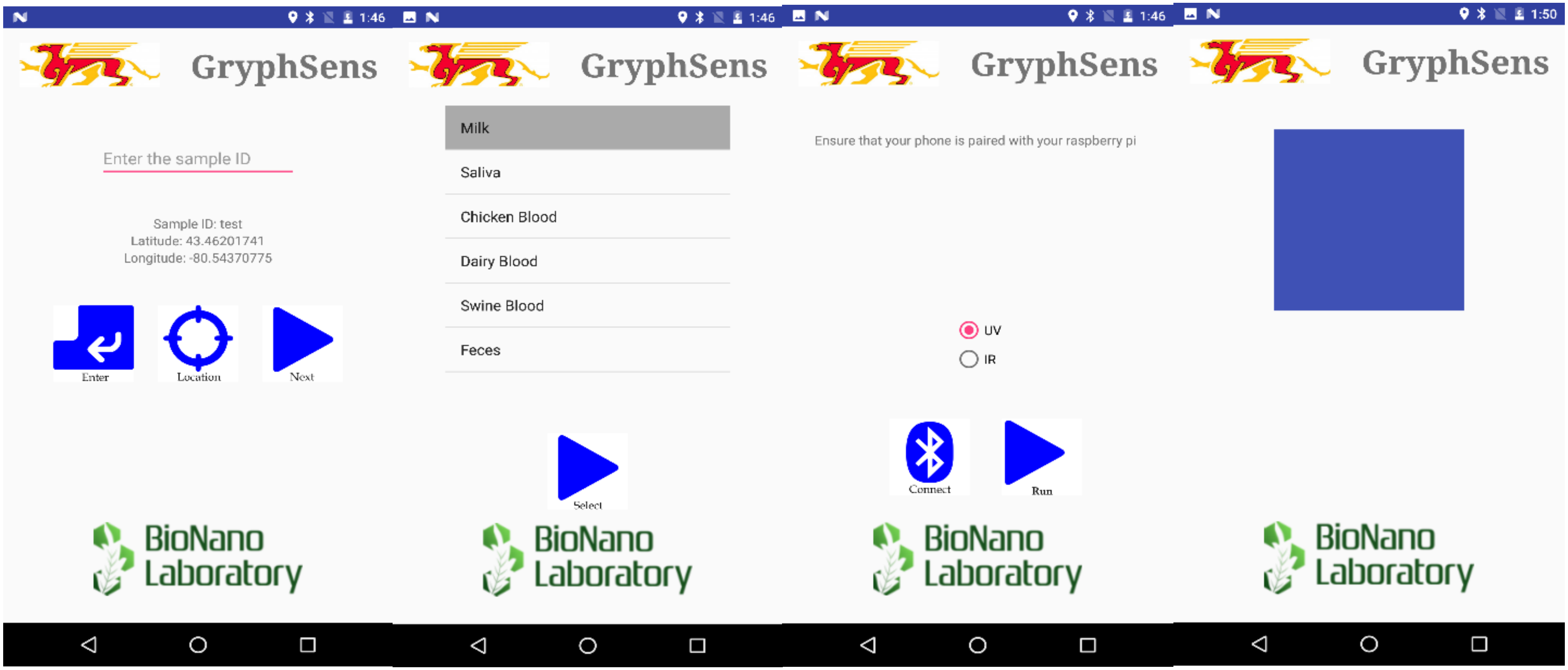

Figure S1. Schematic of the software User Interface (UI) for android smartphones to detect progesterone in milk samples.

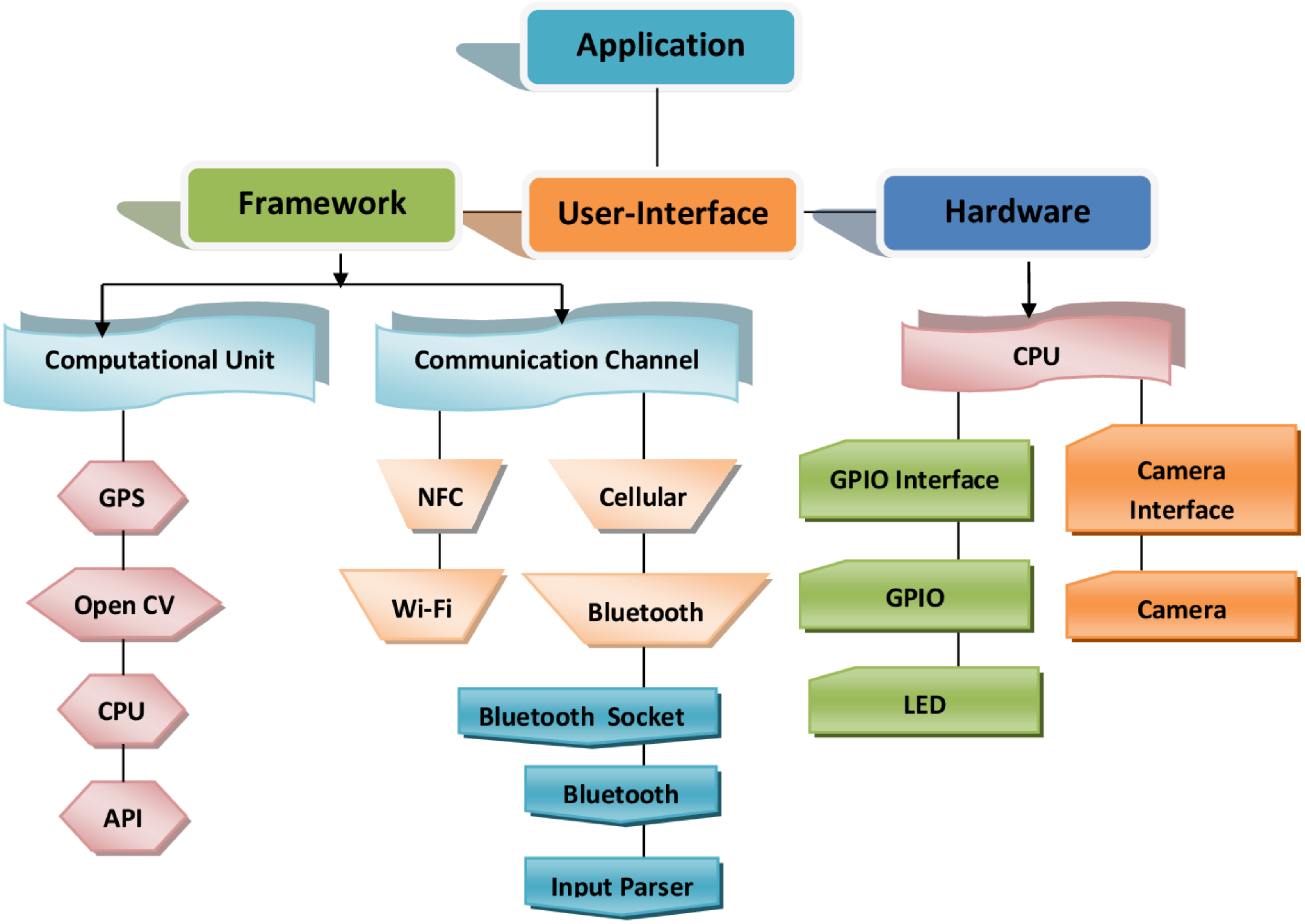

Figure S2. Overall system architecture and design of the portable progesterone detector in the 194 smartphone-controlled biosensor device.

Matrix | Statistics

Data: Spectrum | Edit Matrix | Wavelength: 16 | Show | (450 nm)

	1	2	3	4	5	6	7	8	9	10	11	12
A						1.416	0.422	0.467				
B						1.386	0.316	0.556				
C						1.168	0.113	0.466				
D						0.925	0.092	0.500				
E					0.964	0.970	0.273	0.613				
F					0.699	0.860	0.212	0.678				
G					0.290	0.683	0.318					
H					0.362	0.814	0.329					

	1	2	3	4	5	6	7	8	9	10	11	12
A						Standard 1	Standard 5	Sample 3				
B						Standard 1	Standard 5	Sample 3				
C						Standard 2	Standard 6	Sample 4				
D						Standard 2	Standard 6	Sample 4				
E					Control Low	Standard 3	Sample 1	Sample 5				
F					Control Low	Standard 3	Sample 1	Sample 5				
G					Control High	Standard 4	Sample 2					
H					Control High	Standard 4	Sample 2					

Figure S3. Representative data showing the Optical densities (OD) and plate map of the 96-well plate used in the reader for the Abraxis reference ELISA assay.
